# The ageing holobiont: crosstalk between telomere dynamics, oxidative stress and the gut microbiome

**DOI:** 10.1002/brv.70152

**Published:** 2026-02-27

**Authors:** Michael L. Pepke, Søren B. Hansen, Morten T. Limborg

**Affiliations:** ^1^ Center for Evolutionary Hologenomics, Globe Institute Faculty of Health and Medical Sciences, University of Copenhagen Øster Farimagsgade 5 Copenhagen DK‐1353 Denmark

**Keywords:** dysbiosis, gastrointestinal disease, gut barrier, holobiont, hologenome, microbiota, senescence, telomerase, telomere length, whole genome sequencing

## Abstract

The gut tissue is at the frontline of early onset of ageing. It exhibits high cell turnover rates and rapid telomere shortening, which can have systemic effects on the developing or senescing organism. We conducted a literature review of studies on the crosstalk between telomere length dynamics, telomerase activity, oxidative stress, and gut microbiota composition and activity in animals. Studies mainly on humans and animal models include correlations between telomere dynamics and gut microbiome components, particularly under pathogenic conditions, but also manipulations of either the gut microbiome through faecal microbiota transplantations or of telomere dynamics using telomerase knockout models. This synthesis reveals that components of the gut microbiome including microbial metabolites and pathogenic bacteria can affect telomere dynamics through oxidative‐stress‐inducing processes, and that telomere maintenance is critical in maintaining gut barrier and tissue integrity, which link inflammation and gut dysbiosis. Some of the interactions between the gut microbiome and host telomere dynamics are bidirectional and important in maintaining intestinal homeostasis. However, many of the causal molecular or cellular mechanisms – and how they translate into organismal senescence – remain to be identified. Furthermore, we highlight how recent advances in whole genome sequencing capacities and bioinformatic tools represent an often‐unexploited resource for measuring telomere lengths and may be particularly valuable tools within the hologenomic framework outlined here. Investigating the role of telomere dynamics in mediating gut microbiota–host interactions in different species will improve our understanding of how crosstalk between these hallmarks of ageing shape holobiont physiology in general and the ageing phenotype in particular.

## I. INTRODUCTION

The microbial communities found within the gut of animals play an important role in maintaining host health, modulating immune responses and metabolism (Bordenstein & Theis, 2015; Hou *et al*., 2022). Telomeres are short repetitive DNA sequences protecting the ends of linear chromosomes from degradation and they are highly conserved across eukaryotes (Gomes, Shay & Wright, 2010). Telomeres shorten with cell replication, and their attrition can be accelerated by oxidative damage (von Zglinicki, 2002), triggering cellular senescence when telomeres become too short (Blackburn, Epel & Lin, 2015). Short telomeres are associated with ageing and with higher risk of several diseases in humans (Blackburn *et al*., 2015; Heba *et al*., 2021; Rossiello *et al*., 2022; Wang *et al*., 2018). Recent research indicates that the gut microbiome may influence host telomere dynamics (Chen *et al*., 2022; Mach *et al*., 2017; Velando *et al*., 2021; Xu *et al*., 2023) in particular of the intestinal tissue (El Maï *et al*., 2023). However, the causality underlying associations between gut microbiomes and telomere length (TL) are not well understood, and it has been speculated that telomere dynamics are involved in shaping gut microbiota composition and activity (El Maï *et al*., 2023; Pepke, Hansen & Limborg, 2024a). This may be particularly evident in relation to mechanisms underlying ageing, immunosenescence and inflammation that simultaneously affect many molecular, cellular and intercellular responses and signalling pathways. Here, we identify evidence and knowledge gaps in the potential role of telomere dynamics as mediators of gut microbiota–host interactions. By unravelling how telomere dynamics are involved in microbiota–host interactions we aim to synthesise our understanding of this aspect of host–microbiome interactions and the multifaceted process of ageing, but also of the treatment of gastrointestinal diseases and gut microbiota dysfunction.

The gut tissue has some of the highest telomere shortening rates across tissues in vertebrates (Burraco *et al*., 2023; Carneiro *et al*., 2016; Demanelis *et al*., 2020; Hastie *et al*., 1990). Without maintenance, telomeres are expected to shorten faster in highly proliferative tissues, such as the gut. However, this is not the case in some tissues with high turnover rates such as the gonads and blood of zebrafish (*Danio rerio*), and telomere shortening in the gut may therefore lead to early cellular senescence compared to other organs (Carneiro *et al*., 2016). Telomere biology disorders causing telomere dysfunction are associated with a range of clinical phenotypes including mucosal inflammation and gut barrier disorders (Jonassaint *et al*., 2013; Niewisch *et al*., 2022). In patients with inflammatory bowel diseases (IBD), which leads to gut microbiome dysbiosis (Halfvarson *et al*., 2017; Zhang *et al*., 2022*b*), increased telomere attrition is observed (Chakravarti *et al*., 2021; Risques *et al*., 2008; Sienkiewicz *et al*., 2023*a*). Many diseases are associated with TL, and telomere dysfunction is linked to several gastrointestinal diseases (Allaire *et al*., 2023; Barnes, Fouquerel & Opresko, 2019; LaBella *et al*., 2024; Rudolph, Hartmann & Opitz, 2009; Wang *et al*., 2023). Small‐scale correlational studies in humans have also indicated that leukocyte telomere shortening or TL was associated with predominance of certain bacteria in chronically ill hospitalised patients (Maeda, Horiuchi & Makino, 2021), in patients with severe mental disorders (Manchia *et al*., 2023) and in healthy children (Chen *et al*., 2022). However, inferring causation from correlation requires further insights into the underlying mechanisms of gut tissue telomere dynamics and microbiota functions.

### (1) Ageing of the gut microbiome

Ageing is characterised by progressive organismal deterioration and cellular dysfunction (senescence), which in humans is often accompanied by a range of diseases (Flatt & Partridge, 2018). Ageing is a complex process, and senescent organisms exhibit multifaceted systemic responses including changes in genomic instability, telomere attrition, epigenetic alterations, proteostasis, nutrient‐sensing, mitochondrial function, cellular senescence, stem cell exhaustion, and intercellular communication (López‐Otín *et al*., 2013). These hallmarks of ageing represent molecular or cellular processes that are interconnected, i.e. they are not independent mechanisms of ageing. The hallmarks were recently elaborated and amended by the inclusion of disabled macroautophagy, chronic inflammation – and microbiota dysbiosis (López‐Otín *et al*., 2023).

As the host ages, the gut microbiome gradually shifts, typically towards lower diversity and greater interindividual variation and uniqueness (Conway & Duggal, 2021; Litichevskiy *et al*., 2025; Wilmanski *et al*., 2021). These changes may include increases in harmful bacteria (Ghosh, Shanahan & O'Toole, 2022a; Smith *et al*., 2017), but age‐associated compositional changes can differ between healthy (delayed) and unhealthy (accelerated) ageing phenotypes (Ghosh, Shanahan & O'Toole, 2022b). Loss of microbial functions with age [e.g. microbial biosynthesis, digestion or gut barrier functions (Langille *et al*., 2014; Popkes & Valenzano, 2020)] may lead to dysbiosis that can result in systemic inflammation (Conway & Duggal, 2021; Halfvarson *et al*., 2017; Popkes & Valenzano, 2020). These changes are more strongly associated with an unhealthy than a healthy ageing trajectory (Ghosh *et al*., 2022a,b). Conversely, inflammatory host responses can also drive dysbiosis (Zeng, Inohara & Nuñez, 2017). Dysbiosis broadly refers to an abnormal shift or imbalance in gut microbiome composition and metagenomic function characterised by loss of diversity, commensals and symbionts, and increases in pathobionts (Levy *et al*., 2017; Zeng *et al*., 2017). This can result in disruption of the gut barrier function, which allows microbial components, such as endotoxins (lipopolysaccharides), to translocate from the gut into the bloodstream, triggering an immune response and promoting inflammation and increased levels of reactive oxygen species (ROS) (Hohman & Osborne, 2022; Martel *et al*., 2022; Singh *et al*., 2022). The epithelial cell layer together with the mucosal layer and several immunological products constitute the barrier between the gut microbiota and the host. In addition to transcellular and carrier‐mediated transport, intestinal barrier permeability is regulated by various (paracellular) tight junction protein connections between epithelial cells, which are crucial in maintaining gut homeostasis (Peterson & Artis, 2014). Loss of gut barrier integrity is seen in the pathogenesis of several chronic intestinal diseases including IBD in humans (Chelakkot, Ghim & Ryu, 2018). Increased intestinal permeability during healthy ageing has been observed across several laboratory animal models (Branca, Gulisano & Nicoletti, 2019), and it may be an evolutionarily conserved feature of ageing animals (Salazar *et al*., 2023). However, the evidence that intestinal permeability increases during healthy ageing in humans remains limited (Branca *et al*., 2019).

Chronic inflammation contributes to various age‐related diseases that may involve telomere dynamics (Heba *et al*., 2021; Liu *et al*., 2023a). Similarly, chronic low‐grade inflammation (inflammaging; Franceschi *et al*., 2000), has been associated with accelerated telomere shortening (Jurk *et al*., 2014; Zhang *et al*., 2016). Several molecular and cellular changes linked with ageing contribute to gut dysbiosis (Franceschi *et al*., 2018), prompting the formulation of a ‘hologenome theory of ageing’ (Bana & Cabreiro, 2019). This concept emphasises the interactions between the host and its microbiota in shaping a combined ageing phenotype within the holobiont (*sensu* Bordenstein & Theis, 2015) and is more aptly referred to as the ‘holobiont theory of ageing’ in this review. However, without experimental studies, these interactions remained unverified.

### (2) Telomere dynamics and oxidative stress

Telomeres were originally envisioned as a simple ‘mitotic clock’ (Harley, 1991), but we now know that these short tandem repeats are involved in various dynamic physiological and metabolic processes which result in great heterogeneity in TL dynamics across species, populations, individuals, tissues, cells, and chromosomes (Karimian *et al*., 2024; Lin & Epel, 2022; Pepke, 2024). TL can be a highly plastic trait and respond to oxidative stress‐inducing processes at varying spatiotemporal scales. Telomeres are particularly vulnerable to oxidative stress because of their high guanine content and limited DNA repair mechanism (Barnes *et al*., 2019). Furthermore, oxidative damage can lead to breaks within the telomeres, significantly speeding up their shortening (von Zglinicki, 2002). Oxidative stress results from an imbalance between the production of ROS and antioxidant defence mechanisms, and it provides a functional mechanism linking TL, energy expenditure and metabolism (Casagrande & Hau, 2019; Lin & Epel, 2022; von Zglinicki, Petrie & Kirkwood, 2003). Many of the biological and environmental factors that influence TL – such as reproduction, growth, physical activity, exposure to stressors or disease, diet and abiotic conditions – are often also associated with oxidative stress balance in animals (Chatelain, Drobniak & Szulkin, 2020). A growing body of evidence supports associations between oxidative stress and telomere attrition *in vivo* across different species (Armstrong & Boonekamp, 2023).

Telomerase is a ribonucleoprotein consisting of a telomerase RNA component (TERC), that serves as template for adding telomeric DNA, and a catalytic protein component, telomerase reverse transcriptase (TERT), that synthesises DNA in humans (Blackburn *et al*., 2015). Telomerase can thus maintain or elongate telomeres, and is active in most stem, germ and cancer cells, but suppressed in somatic tissues of many endotherms (e.g. mammals and birds) although less often in ectotherms (e.g. fish, reptiles and invertebrates; Gomes *et al*., 2010).

Certain gut bacteria can contribute to local oxidative damage by modulating mitochondrial activity and through the production of ROS or pro‐oxidant microbial metabolites (Jones, Mercante & Neish, 2012; Shi *et al*., 2022), which can result in inflammation with systemic consequences (Al‐Daghri *et al*., 2021; Franceschi *et al*., 2018). However, local microbial‐elicited ROS production by host intestinal epithelial cells may be established as a host immune response to pathogens (Aviello & Knaus, 2018; Jones *et al*., 2012). Furthermore, ROS production by host cells may be induced by microbial signals to contribute to the physical separation of host tissues from luminal microbial communities, thereby reinforcing gut barrier integrity and sustaining intestinal homeostasis (e.g. Bai *et al*., 2025). Conversely, other gut bacteria can reduce oxidative stress through antioxidative metabolites such as short‐chain fatty acids (SCFAs) (Dam, Misra & Banerjee, 2019). Furthermore, a healthy and diverse microbiome may prevent colonisation by pathogens (Spragge *et al*., 2023) that could increase telomere attrition (Chatelain *et al*., 2020). However, telomere dysfunction may in turn increase susceptibility to bacterial pathogens (Kang *et al*., 2018), suggesting a possible bidirectional relationship. Oxidative stress may consequently play a central role in crosstalk between the gut microbiome and intestinal telomere and telomerase dynamics.

### (3) Literature search

**Fig. 1:**
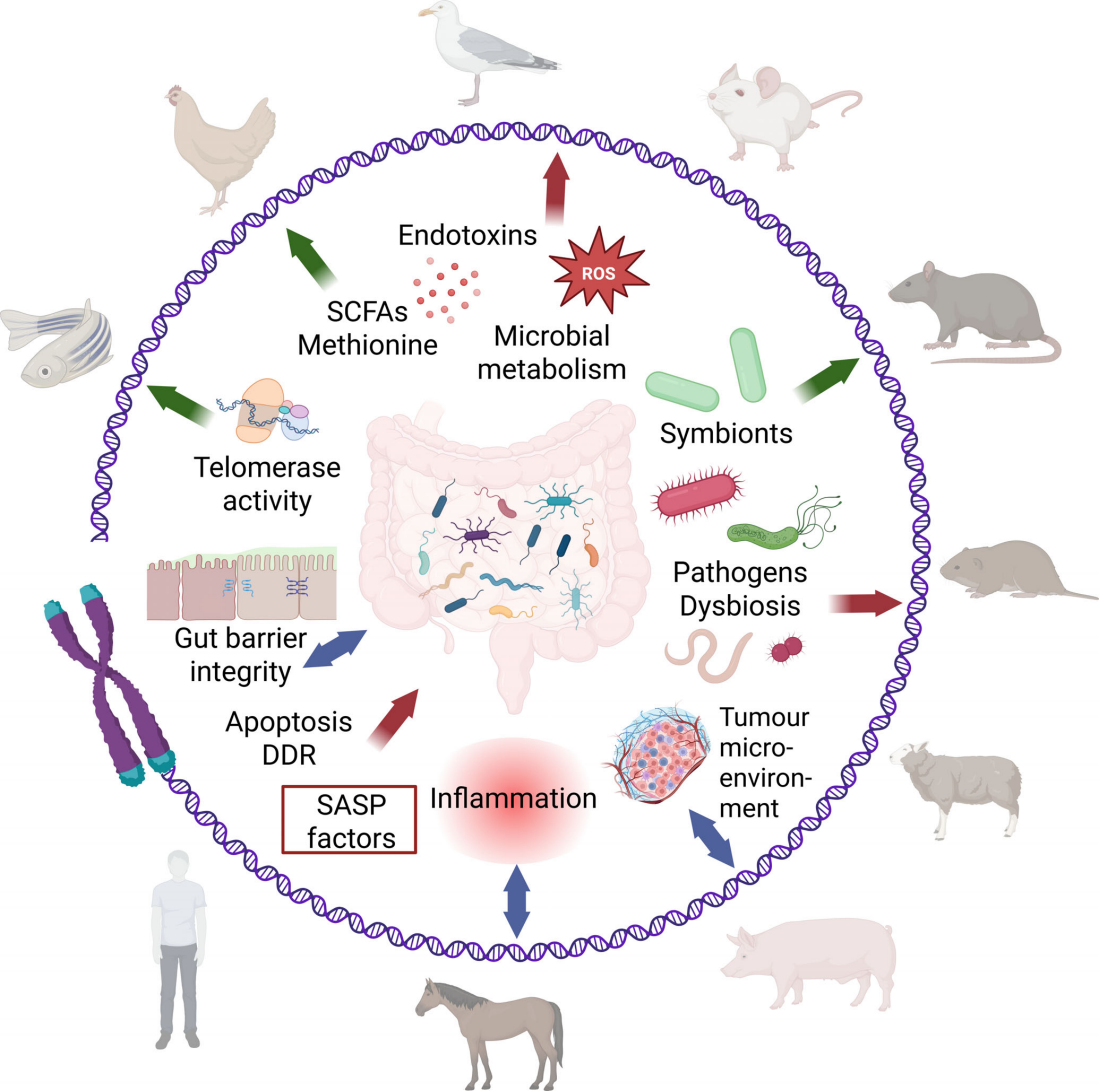
Examples of components and mechanisms underlying associations and interactions between the gut microbiome and host telomere dynamics and the species in which these interactions have been investigated (see Table 1). Coloured arrows indicate primarily positive (green) or negative (red) effects on telomere length while bidirectional interactions are marked in blue. DDR, DNA damage response; ROS, reactive oxygen species; SASP, senescence‐associated secretory phenotype; SCFA, short‐chain fatty acid.

To obtain an overview of research on the associations between the gut microbiome and host telomere dynamics, and to identify knowledge gaps, we conducted a scoping review (Munn *et al*., 2018) of primary literature. We first performed a comprehensive systematic literature search using the *Web of Science* (Clarivate) and *Scopus* (Elsevier) databases (date of last search: July 7th 2025) for abstracts, titles and key words of peer‐reviewed articles (excluding reviews, opinions, perspectives, etc.) in English containing the following terms: telomer* AND microbi* AND (‘gut’ OR ‘intest*’ OR ‘gastro*’ OR ‘faecal’ OR ‘fecal’). We then performed forward and backward reference searches of the identified studies, and we searched the two databases in addition to *Google Scholar* for gut physiology studies utilising telomerase knockout models, and for specific gastrointestinal diseases (e.g. IBD) investigated in relation to telomere biology. Studies were individually screened to assess whether they (*i*) included analyses of both the gut microbiome and host telomere dynamics in any tissue, (*ii*) were based on empirical data collected *in vivo* or *in vitro*, and (*iii*) reported how and where TL was measured. All cohorts of animals in the studies were included across different ages. Studies that only reported simultaneous changes in TL and gut microbiomes with age without linking these were not included, but experimental treatments affecting both were included in a separate category. We included studies measuring TL using different methodologies (discussed in Section I.4) in various tissues (mainly blood or intestinal tissues), because TLs are often found to be correlated across tissues within an organism and are largely congruent across methodologies (Demanelis *et al*., 2020; Lai, Wright & Shay, 2018; McLester‐Davis *et al*., 2023). We included studies that use faecal samples as a proxy to profile the gut microbiota, which predominantly reflects the microbiota of the distal colon and may not accurately capture the composition or activity of microbial communities in the small intestine, where much of the nutrient absorption and host–microbiota interaction occurs (Shalon *et al*., 2023). We collectively refer to the ‘gut microbiome’ irrespective of the spatial origin within the intestinal environment. The studies were then sorted *a posteriori* into the topics: (*i*) associations between telomere dynamics and the gut microbiome, (*ii*) telomere dynamics and faecal microbiota transplantation, (*iii*) gut microbiomes of telomerase knockout models, (*iv*) telomere dynamics under gastrointestinal pathogenic conditions, and (*v*) experimental treatments affecting both telomere and microbiota dynamics. The resulting 79 studies were sorted according to species. An overview of these studies is provided in Table 1 and Fig. 1. The literature search process is shown in a PRISMA diagram in Fig. S1 in the online Supporting Information.

**Table 1:** Overview of the 79 empirical studies that investigated interactions or associations between telomere length (TL) or telomerase dynamics, the gut barrier, and components of the gut microbiome and intestinal lumen. Studies that report the same general results are combined in rows. *In vivo* and *in vitro* studies included human, mouse (*Mus* spp.), brown (laboratory) rat (*Rattus norvegicus*), pig (*Sus domesticus*), sheep (*Ovis aries*), domestic horse (*Equus ferus caballus*), Brandt's vole (*Lasiopodomys brandtii*), zebrafish (*Danio rerio*), chicken (*Gallus gallus domesticus*), and yellow‐legged gull (*Larus michahellis*).

Species	Main findings	Reference
Associations between telomere dynamics and the gut microbiome		
Yellow‐legged gull	TL was associated with gut microbiota composition including taxa (e.g. *Cetobacterium*, *Catellicoccus*) influencing oxidative stress balance.	Velando *et al*. (2021)
Domestic horse	TL was associated with gut microbiota composition including SCFA‐producing taxa (e.g. Firmicutes) in foals.	Mach *et al*. (2017)
Domestic horse	No association between TL and gut microbiota composition in adult horses.	Plancade *et al*. (2019)
Pig	Genes enriched for telomere maintenance functions were associated with commensal *Subdoligranulum* bacteria and upregulated in colons of pigs fed with a rapeseed‐meal‐based feed that may increase oxidative stress.	Onarman Umu *et al*. (2024)
Brown rat	TL positively associated with SCFA concentration in the colon.	O'Callaghan *et al*. (2012)
Mouse	The abundances of several gut bacteria were positively (e.g. Pseudomonadaceae) or negatively (e.g. Coriobacteriaceae) associated with brain telomerase levels.	Liu *et al*. (2019a)
Human	Individuals born in a ‘higher microbial exposure season’ had shorter adult TL (no microbiome data).	Tennyson *et al*. (2018)
Human	TL was negatively associated with abundances of several members of Firmicutes including Lachnospiraceae (including *Ruminococcus torques*, *Lachnoclostridium phocaeense*), but not with faecal SCFAs in children.	Chen *et al*. (2022)
Human	Interaction between TL and *Lachnoclostridium* abundance predicted major depressive disorder.	Manchia *et al*. (2023)
Human	TL was negatively associated with gut‐derived endotoxin (LPS) in blood.	Al‐Daghri *et al*. (2021); Al‐Daghri *et al*. (2022)
Human	TL estimated from DNA methylation profiles was negatively associated with bacterial taxa enriched (e.g. *Catenibacterium*, *Prevotella*, *Alloprevotella*) and positively associated with taxa depleted (e.g. *Alistipes*, *Subdoligranulum*, *Coprococcus*, *Faecalibacterium*) in people with HIV. TL was associated positively with tight junction integrity (ZO‐1) and negatively with microbial translocation markers (e.g. LPS binding protein).	Singh *et al*. (2024)
Human	TL in blood was negatively associated with gut microbial antibodies, including *Bacteroides* fimbrillin.	Andreu‐Sánchez *et al*. (2024)
Human	TL and cytokine responses were negatively correlated with the gut microbial cometabolite hippurate (which is associated with microbiota diversity) in males but not females.	Bulut *et al*. (2024)
Human	Mendelian randomisation analysis found positive genetic associations between TL and *Bacteroides salyersiae* and *Bifidobacterium longum* and negative associations with *Eubacterium rectale*, *Anaerotruncus*, and *Holdemania*.	Zhang *et al*. (2025b)
Telomere dynamics and faecal microbiota transplantation (FMT)		
Brandt's vole	Brain TL was associated with gut microbiota composition. FMT from high‐density environments led to shorter telomeres.	Xu *et al*. (2023)
Mouse	FMT from young to old mice did not affect blood TL, but there were no TL differences between young and old mice.	Boehme *et al*. (2021)
Mouse	FMT from young to old mice did not affect brain TL. TL was shorter in old mice.	Lin *et al*. (2024a)
Mouse	TL and telomerase activity in intestines and aortas increased after FMT from young to old mice and decreased after FMT from old to young mice. TL was shorter in old mice.	Cheng *et al*., (2024); Cheng *et al*. (2025)
Gut microbiomes of telomerase knockout (KO) models		
Mouse and zebrafish	Villi defects, degeneration of the intestinal epithelia and/or gut barrier dysfunction found in telomerase KO mice and zebrafish, which may be restored by telomerase reactivation.	Chen *et al*. (2015); El Maï *et al*. (2023); Ellis *et al*. (2022); Hao *et al*. (2005); Henriques *et al*. (2013); Herrera *et al*. (1999); Jaskelioff *et al*. (2011); Rudolph *et al*. (1999); Şerifoğlu *et al*. (2024); Smoom *et al*. (2023); Tomás‐Loba *et al*. (2008); Zhang *et al*. (2025a)
Mouse and zebrafish	Short telomeres activated YAP1 causing IBD, which was ameliorated by gut telomerase reactivation. Increased expression of YAP target genes in telomerase KO zebrafish.	Chakravarti *et al*. (2020); Chakravarti *et al*. (2021); El Maï *et al*. (2023)
Zebrafish	Telomerase‐deficient fish had lower microbial diversity and dysbiosis, higher methionine levels, and increased expression of SASP factors, which was restored by telomerase reactivation. Telomerase KO fish had decreases in Alphaproteobacteria and Planctomycetes and increases in Gammaproteobacteria, Bacteroidia and Fibrobacteria.	El Maï *et al*. (2023)
Zebrafish	Telomere shortening triggered intestinal secretion of SASP factors.	Lex *et al*. (2020)
Zebrafish	Telomerase KO fish had reduced phagocytosis of *Escherichia coli* and increased gut permeability, DNA damage response markers and apoptosis.	Ellis *et al*. (2022)
Mouse	Antibiotic treatment of telomerase KO mice reduced intestinal inflammation.	Chakravarti *et al*. (2020)
Mouse	Telomerase KO mice with short telomeres had reduced gut microbiota diversity, compromised gut barrier permeability and increased expression of inflammatory markers. Gut microbiota was enriched in Actinobacteria.	Liu *et al*. (2023b)
Mouse	Telomerase KO mice had lower abundance of *Bifidobacterium adolescentis* and impaired gut barrier. Heat‐inactivated *B. adolescentis* supplementation improved barrier integrity.	Qi *et al*. (2023)
Mouse	Telomerase deficiency (KO) enhanced susceptibility to *Helicobacter*‐induced IBD.	Eaton *et al*. (2011)
Telomere dynamics under gastrointestinal pathogenic conditions		
Mouse and human	*Helicobacter pylori* infection was associated with shorter telomeres leading to gut inflammation.	Aslan *et al*. (2013); Kuniyasu *et al*. (2003); Lee *et al*. (2016); Wu *et al*. (2019)
Mouse	*Salmonella enterica* infection caused leukocyte telomere attrition in wild‐derived mice. Mice with longer initial TL were more resistant to the infection.	Ilmonen *et al*. (2008)
Soay sheep	TL was *positively* associated with gastrointestinal strongyle nematode parasite burden, but not with helminth‐specific antibody levels, in feral sheep.	Ravindran *et al*. (2024)
Human	TL was shorter in subjects with a gastrointestinal helminth infection compared to uninfected controls.	Macamo *et al*. (2024)
Human	Telomerase activity decreased significantly after *H. pylori* eradication.	Chung *et al*. (2002)
Human	Increased telomere shortening in patients with IBD.	Friis‐Ottessen *et al*. (2014); Kinouchi *et al*. (1998); O'Sullivan *et al*. (2002); Risques *et al*. (2008); Risques *et al*. (2011); Tahara *et al*. (2015); Watanabe *et al*. (2022)
Human	No association between telomere shortening and IBD.	Getliffe *et al*. (2005)
Human	Humans with defects in telomere maintenance genes are more likely to suffer from IBD. In organoids, telomere dysfunction promoted inflammation, and telomerase activation reduced inflammation.	Chakravarti *et al*. (2021); Jonassaint *et al*. (2013)
Human	Potential relationships between TL and IBS and GERD.	Wang *et al*. (2023)
Human	No association between TL and sCD14 (a marker of gut permeability) in HIV‐infected children, which have shorter TL and increased gut microbial translocation compared to uninfected children.	Gianesin *et al*. (2016)
Human	No association between mucosa‐associated microbiome diversity and oesophageal TL in patients with Barrett's oesophagus.	Shijimaya *et al*. (2024)
Human	In chronically ill patients, shorter methylated subtelomere length was associated with increased non‐pathogenic *E. coli* or *Streptococcus*.	Maeda *et al*. (2021)
Human	Higher expression of a set of telomere‐related genes was associated with intratumoral microbiome profiles of colorectal cancer patients including higher abundance of pathogenic *Selenomonas* and *Lachnoclostridium*.	Zhang *et al*. (2025c)
Experimental treatments affecting telomere and microbiota dynamics		
Chicken	No effect of diet supplementation with the presumed probiotic *Bacillus subtilis* on blood telomere shortening in laying hens.	Makarenko *et al*. (2019)
Chicken	Broiler chickens fed with corticosterone had shorter telomeres and smaller intestinal villi height and crypt depth inflammation (no microbiome data).	Badmus *et al*. (2021)
Mouse	Young mice responded to *E. coli*‐derived LPS by increasing expression of proteins involved in TL maintenance, which was impaired in old mice.	Criscuolo *et al*. (2018)
Mouse	Simultaneous upregulation of gut telomerase expression and restoration of gut dysbiosis in old mice treated with an herbal extract.	Shenghua *et al*. (2020)
Mouse	Culture supernatants of bacterial metabolites from *Streptococcus thermophilus* reduced telomere attrition in blood and liver of D‐galactose‐induced aged mice, but no effect of *Lactobacillus rhamnosus* was observed.	Shan *et al*. (2020)
Mouse	Icariin treatment altered gut microbiota composition and upregulated the telomere binding protein Pot1α, similar to the effects of FMT from icariin‐treated mice.	Li *et al*. (2021)
Mouse	Ginseng treatment increased brain TL, but FMT from ginseng‐treated mice did not affect TL.	Lin *et al*. (2024b)
Mouse	Ginseng increased levels of protein components of TERT and of tight junctions in old mice and changed gut microbiota composition.	Lei *et al*. (2022b)
Mouse	Mealworm protein diet increased liver TL and gut microbial diversity and reduced the abundance of some pathogenic bacteria in D‐galactose‐induced aged mice.	Anusha & Negi (2024)
Mouse and human	NMN administration reduced microbiota diversity, altered relative abundances of bacteria and increased TL.	Niu *et al*. (2021)
Brown rat	Blood TLs were longer in D‐galactose‐induced aged rats fed with probiotic *Limosilactobacillus fermentum*.	Lee *et al*. (2023)
Brown rat	Simultaneous increase in brain telomerase levels and changes in gut microbiome composition in D‐galactose‐induced aged rats treated with partially hydrolysed guar gum.	Liu *et al*. (2019b)
Brown rat	Exposure to polychlorinated biphenyls reduced telomerase activity and downregulated telomere maintenance genes in bone marrow cells – and altered the gut microbiota indicating dysbiosis.	Wang *et al*. (2020)
Brown rat and human	Administration of *Lactobacillus* (or ‘nutraceutical supplements’ containing *Lactobacillus*) reduced telomere shortening or increased TL.	Hor *et al*. (2019); Lew *et al*. (2019); Tsoukalas *et al*. (2019)
Human	Colonic organoids derived from patients with ulcerative colitis treated with an herbal extract increased telomerase activity and TL, and reduced inflammation.	Watanabe *et al*. (2022)
Human	Comparing astronaut twins, a one‐year spaceflight reversibly increased TL and altered gut microbiome composition including microbial metabolites.	Garrett‐Bakelman *et al*. (2019)
Human	Telomerase was upregulated by treating endothelial cells with the SCFA acetate *in vitro* counteracting presumed telomere shortening by the gut bacterial metabolite phenylacetic acid.	Saeedi Saravi *et al*. (2025)
Human	Blood telomere shortening decreased in humans fed a multistrain probiotic supplementation containing *Lactobacillus* spp., *Bifidobacterium* spp., *Streptococcus thermophilus*, and *Saccharomyces boulardii*.	Chaithanya *et al*. (2025)

GERD, gastroesophageal reflux disease; HIV, human immunodeficiency virus; IBD, inflammatory bowel disease; IBS, irritable bowel syndrome; LPS, lipopolysaccharide; NMN, nicotinamide mononucleotide; Pot1α, protection of telomeres 1α; SASP, senescence‐associated secretory phenotype; SCD14, soluble cluster of differentiation 14; SCFA, short‐chain fatty acid; TERT, telomerase reverse transcriptase; YAP1, yes‐associated protein 1; ZO‐1, zonula occludens‐1.

### (4) Telomere length measurement techniques

The studies reviewed here employed several different laboratory‐based methods for estimating TL. These methods, which have been developed during the last four decades, differ in their abilities to measure the mean, distribution, chromosome‐specific, absolute or relative TLs (Lai *et al*., 2018; Yu, Byun & Park, 2024). The application of quantitative PCR (qPCR) to telomeric DNA to determine average TL relative to the amount of a single copy gene (Cawthon, 2002) has allowed cost‐effective high‐throughput studies of small amounts of DNA, which has become particularly popular in ecology and large‐cohort studies. Terminal restriction fragment (TRF) length analysis, in which a distribution of TLs is determined after digesting non‐telomeric DNA with restriction enzymes (Kimura *et al*., 2010), is often referred to as the standard TL measurement technique due to its early development and high accuracy. Average TL is measured in most studies discussed in this review, but it may often be the shortest telomeres that are most important to telomere dysfunction and activation of DNA damage responses (DDRs) (Hemann *et al*., 2001; Lai *et al*., 2018). Furthermore, there are several issues with comparing TL measurements directly across studies and laboratories due to e.g. differences in study‐specific reference samples and inclusion of interstitial telomeres (i.e. non‐functional telomeric sequences found within chromosomes, which are included in qPCR measurements and some TRF methodologies; Lindrose *et al*., 2021), or differences in the range of molecular weight markers and inclusion of subtelomeric regions (for TRF, see Kimura *et al*., 2010; Pepke, Ringsby & Eisenberg, 2023b). This has largely hindered cross‐study analyses including interspecific comparisons of absolute TLs (Pepke, 2024). Other methods to quantify absolute TLs include fluorescence *in situ* hybridisation (FISH) or whole genome sequencing (WGS) (discussed in Section VI).

## II. CROSSTALK BETWEEN INTESTINAL TELOMERE DYNAMICS AND THE GUT MICROBIOME

### (1) Associations between telomere dynamics and the gut microbiome

Several explorative studies have investigated associations between telomere dynamics and the gut microbiome in different vertebrate species. Velando *et al*. (2021) found that erythrocyte telomeres were longer in wild yellow‐legged gulls (*Larus michahellis*) with gut microbiotas enriched in members of *Cetobacterium* and *Catellicoccus* species. The antioxidant vitamin B12 is produced by *Cetobacterium*, which can therefore potentially affect TL (Li *et al*., 2022; Praveen, Sivaprasad & Reddy, 2022). *Catellicoccus* contributes to reducing oxidative stress through several enzymatic functions akin to the effects of symbiotic *Lactobacillus* (*L. fermentum*, *L. paracasei*, *L. helveticus*, *L. plantarum*, and *L. reuteri*) reducing telomere shortening in brown rats (*Rattus norvegicus*) (Hor *et al*., 2019; Lew *et al*., 2019) although no such effects were observed with *L. rhamnosus* in mice (*Mus musculus*) (Shan *et al*., 2020). Mach *et al*. (2017) found that leukocyte telomeres were longer in foals of domestic horses (*Equus ferus caballus*) with gut microbiotas enriched with butyrate‐producing Firmicutes bacteria (including *Eubacterium*, *Lactobacillus*, *Coprococcus*, *Clostridium* cluster XI, and *Blautia* spp.) and with higher butyrate concentrations. Butyrate, a key SCFA, can mitigate oxidative stress in the human and rodent gut (Hamer *et al*., 2009; Xu *et al*., 2023) for example by suppression of the nuclear factor kappa‐light‐chain‐enhancer of activated B cells (NF‐κB) signalling pathway, which is a key regulator of inflammation (Bian *et al*., 2023). However, the same pattern was not evident in adult horses (Plancade *et al*., 2019) and a butyrate treatment did not affect TL in Brandt's voles (*Lasiopodomys brandtii*) (Xu *et al*., 2023). Higher concentrations of the most common SCFAs (butyrate, acetate and propionate) in the colon of brown rats has similarly been associated with increased colonocyte TL (O'Callaghan *et al*., 2012) and acetate upregulated telomerase in human endothelial cells *in vitro* (Saeedi Saravi *et al*., 2025). However, no correlation was found between total concentration of 10 faecal SCFA subtypes and leukocyte TL in humans (Chen *et al*., 2022). Thus, the mechanisms driving associations between SCFAs and telomere dynamics remain unclear due to conflicting results.

In humans, blood TL or telomere attrition has been negatively correlated with gut microbial‐specific antibodies (Andreu‐Sánchez *et al*., 2024) and with gut‐derived endotoxin (lipopolysaccharide, LPS) (Al‐Daghri *et al*., 2021, 2022), which is known to induce oxidative stress in animal models (Sakaguchi & Furusawa, 2006). Human blood TL has also been negatively associated with markers of increased microbial translocation (Singh *et al*., 2024) and with specific microbial taxa including proinflammatory *Catenibacterium* and *Prevotella* (Singh *et al*., 2024), *Ruminococcus torques*, *Lachnoclostridium phocaeense* and other members of Lachnospiraceae (Chen *et al*., 2022) including *Eubacterium rectale* (Zhang *et al*., 2025b). *R. torques* can degrade mucus of the gut barrier and is associated with IBD (Schaus *et al*., 2024). *E. rectale* is abundant in the human gut and produces butyrate, but, like other Gram‐negative bacteria it also produces LPS (Wang *et al*., 2021a). Thus, expansion of *E. rectale* may lead to elevated LPS production, which ultimately promotes inflammation and IBD (Wang *et al*., 2021a), exemplifying the dual role of many amphibiotic gut bacteria.

The Mendelian randomisation study by Zhang *et al*. (2025b) also revealed positive associations between TL and *Bacteroides salyersiae*, which may be important for healthy ageing in humans (Wilmanski *et al*., 2021), and the probiotic *Bifidobacterium longum*, which can be used in IBD treatment (Yao *et al*., 2021). Singh *et al*. (2024) found positive associations between TL estimated from DNA methylation profiles (DNAmTL) and tight junction integrity indicating intestinal permeability and with SCFA‐producing taxa including e.g. *Alistipes*, *Subdoligranulum*, *Coprococcus*, and *Faecalibacterium* in a study comparing subjects living with and without human immunodeficiency virus (HIV). In pigs (*Sus domesticus*) fed with a rapeseed‐based feed that may increase oxidative stress through a metabolic response (Chen *et al*., 2018), genes enriched for telomere maintenance functions were upregulated (Onarman Umu *et al*., 2024). Furthermore, the gut microbiota of rapeseed‐fed pigs had higher abundances of commensal *Subdoligranulum* bacteria, which were positively associated with telomere‐maintenance‐related genes (Onarman Umu *et al*., 2024). This suggests a dynamic regulation of telomere maintenance in response to changes in intestinal oxidative stress and the gut microbiome, but neither TL nor oxidative stress were measured in their study. *Subdoligranulum* has also been associated with longer DNAmTL in humans (Singh *et al*., 2024).

These correlational studies suggest that the activity of the gut microbiome can affect telomere dynamics and that certain microbial taxa and their metabolites are associated with TL (see Table 1). However, to investigate further the direction of such associations, experimental approaches are needed. Telomere–microbiota interactions may be probed by manipulating the gut microbiome (Section II.2) or host telomere dynamics (Section II.3).

### (2) Telomere dynamics and faecal microbiota transplantation

Faecal (or intestinal) microbiota transplantation (FMT) is a procedure where faecal matter is transferred from a donor to the gastrointestinal tract of a recipient to investigate how changes in the gut microbiome influence host physiology (Bárcena *et al*., 2019). Xu *et al*. (2023) showed that transplanting faeces from Brandt's voles housed under stressful high crowding‐density conditions led to significantly shorter telomeres (measured in the brain) in individually housed recipient voles. Receiving FMTs from individuals housed under high density also resulted in higher corticosterone levels and altered gut microbiotas, including increased abundance of *E. rectale* (see Section II.1) and decreased abundance of the probiotic *Bacteroides uniformis* (Xu *et al*., 2023), which has also been associated with gut telomerase deficiency in zebrafish (El Maï *et al*., 2023). Voles that were housed under high density had shorter telomeres, and their gut microbiota was enriched with members of the genus *Alistipes* (which may be pathogenic under certain conditions; Parker *et al*., 2020) compared to voles housed under low density, which had enriched abundance of *Akkermansia* (*A. muciniphila* has the potential to increase lifespan in a progeroid mouse model; Bárcena *et al*., 2019). However, the FMT changes did not directly recapitulate the gut microbiotas of the donors (e.g. changes in *Alistipes* and *Akkermansia*). Furthermore, it may be difficult to separate effects of crowding and social stress, e.g. social Brandt's voles are not normally individually housed. Yet, this study indicates that environmental stress factors can influence TL (e.g. Chatelain *et al*., 2020) indirectly through changes in the gut microbiota, but it remains unknown how the gut microorganisms or their metabolites affected TL. For example, enrichment of the genus *Alistipes*, which can produce SCFAs, is associated with longer DNAmTL in humans (Singh *et al*., 2024).

In the exceptionally short‐lived African turquoise killifish (*Nothobranchius furzeri*), Smith *et al*. (2017) showed that transferring intestinal content from young to old fish delayed age‐dependent locomotor activity decline and extended lifespan. Whether TL changes were involved in mediating these effects was not assessed, but in killifish, TL shortens with age (Hartmann *et al*., 2009) and may predict lifespan (Reichard *et al*., 2022). Boehme *et al*. (2021) found that FMT from young mice into aged mice reversed age‐associated changes in brain immunity and improved cognitive behaviour, but without significantly altering TL. However, contrary to expectations, there was also no difference in TL between young and old mice, suggesting that laboratory mice may show atypical telomere dynamics (discussed in Section III).

We found no studies testing the effect of autologous FMT (i.e. FMT of samples collected at a younger age from the same individual and stored) on telomere dynamics, although such designs may reduce confounding host effects of the FMT and reveal interactions associated with ageing (Ke, Weiss & Liu, 2022). While it can be difficult to disentangle and control the effects of FMTs consisting of many different metabolites and microorganisms, future functional studies may use host–microbe co‐cultures (gut‐on‐a‐chip systems) to simulate the responses of gut tissue to different microbiotas *in vitro* (Aizpurua *et al*., 2023). Another innovative approach is targeted changes in the abundance of specific gut bacteria using clustered regularly interspaced short palindromic repeats (CRISPR)/CRISPR‐associated protein (Cas) systems to induce mutant bacteria with reduced growth (Beller *et al*., 2023). Beller *et al*. (2023) used mice colonised with a few selected genome‐sequenced bacterial strains, and the approach is currently not applicable to diverse, natural microbiomes. However, in future studies, such knockdown of specific bacteria *in vivo* provides a targeted approach to test hypotheses regarding the absence of specific taxa on telomere dynamics, which ultimately may better resemble natural fluctuations in microbial communities.

### (3) The role of telomere maintenance in host–microbiome interactions

Telomerase knockout organisms, in which telomerase is genetically inactivated or reduced, have been used to study the consequences of progressive telomere shortening and dysfunction in several animal models. In old telomerase (*Terc*) knockout mice, Rudolph *et al*. (1999) and Herrera *et al*. (1999) reported villi defects (atrophy) associated with body mass loss presumably due to decreased nutrient absorption. Accordingly, telomerase (*Tert*) reactivation has been shown to restore villi and reduce intestinal apoptosis in other mouse studies (Jaskelioff *et al*., 2011; Tomás‐Loba *et al*., 2008). Chen *et al*. (2015) found severe degeneration of the intestinal epithelia including villi defects, mucosal ulceration, infection and inflammation (colitis), while no histological changes were observed in the liver, lung or spleen of old telomerase (*Terc*) knockout mice, similar to what has been observed in telomerase (*tert*) knockout zebrafish (Ellis *et al*., 2022; Henriques *et al*., 2013; Şerifoğlu, Lopes‐Bastos & Ferreira, 2024). Preliminary results have similarly indicated that telomere shortening directly impairs gut cell (enterocyte) differentiation in telomerase (*Tert*) knockout mice resulting in gut barrier disruption, repression of tight junction components, microvilli loss and nutrient malabsorption, which induced colitis and became worse as the mice aged (Engevik *et al*., 2022).

It is also possible to manipulate TL without genetic alterations and generate mice with very long telomeres *via in vitro* expansion of embryonic stem cells derived from the inner cell mass (Varela *et al*., 2016). Such mice showed increased lifespan and health (Muñoz‐Lorente, Cano‐Martin & Blasco, 2019), which may be due to direct anti‐inflammatory effects of telomeric DNA (see Bonafè, Sabbatinelli & Olivieri, 2020; Kang *et al*., 2018).

Telomerase (*tert*) knockout zebrafish had shorter telomeres, reduced microbial diversity and a dysbiotic microbiota community characterised by increases in some pathogenic Bacteroidia (e.g. *Bacteroides*) and Gammaproteobacteria (e.g. *Aeromonas* and *Shewanella*), and reductions in some commensal or beneficial Alphaproteobacteria (e.g. *Reyranella* and *Defluviimonas*; El Maï *et al*., 2023). Restoring intestinal telomerase expression led to higher gut microbiota diversity, restored microbiota composition, and reduced DNA damage and gut tissue dysfunction. The telomerase‐deficient zebrafish had increased levels of the essential amino acid methionine in the gut. In humans, methionine levels are negatively correlated with leukocyte TL (van der Spek *et al*., 2019) and age‐associated dysregulation of the methionine cycle has been reported (Parkhitko *et al*., 2019). Methionine restriction extends lifespan in yeast, worms, flies, mice, rats and human cells (Parkhitko *et al*., 2019). This involve decreases in oxidative damage through induction of autophagy, decreased ROS production, and increased antioxidative hydrogen sulfide production (Kitada *et al*., 2021), which we speculate could lead to increases in TL.

**Fig. 2:**
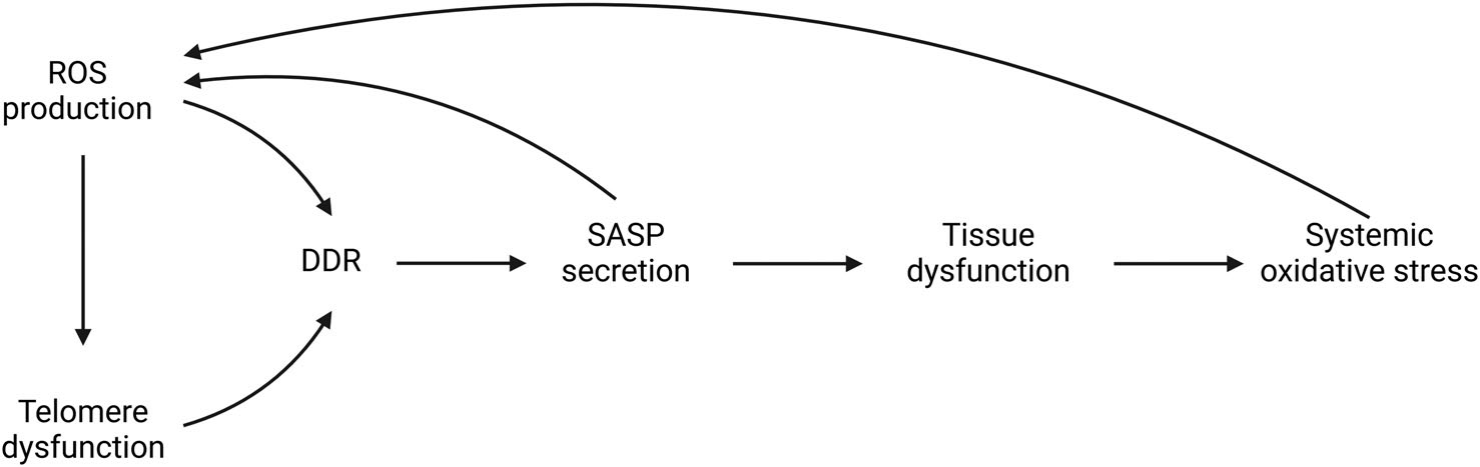
Simplified diagram showing a general feedback loop of telomere dysfunction (i.e. telomere shortening and damage, uncapping, maintenance dysfunction, telomerase dysfunction, etc.) accelerating DNA damage response (DDR), senescence‐associated secretory phenotype (SASP) factor secretion and local tissue dysfunction in response to reactive oxygen species (ROS) production, resulting in systemic oxidative stress.

The telomerase‐deficient zebrafish also had increased expression of senescence‐associated secretory phenotype (SASP)‐related genes (El Maï *et al*., 2023). SASP factors include inflammatory cytokines, chemokines, hydrogen peroxide, growth factors, and oxidised lipids, which are secreted by senescent cells (Kawamoto & Hara, 2024; López‐Otín *et al*., 2013). Telomere dysfunction activates DDR which promotes the release of SASPs in senescent cells (Rossiello *et al*., 2022). Lex *et al*. (2020) found that telomerase (*tert*) knockout zebrafish with short telomeres had increased expression of senescence markers in the intestine and increased systemic inflammation, which was likely caused by SASP factor secretion triggered by telomere shortening. This is presumably part of a feedback loop (Fig. 2) where telomere dysfunction enhances ROS production and oxidative stress, which further facilitates DDR, SASP factor secretion and tissue dysfunction (von Zglinicki, 2024). In the gut, this loop appears to have a disproportionate effect on systemic oxidative stress (Sienkiewicz *et al*., 2023a). Furthermore, both short telomeres (Şerifoğlu *et al*., 2025), dysbiosis and intestinal mucosal damage (Dimitrov *et al*., 2025) can activate and interact with the cyclic GMP‐AMP synthase (cGAS)‐stimulator of interferon genes (STING) innate immune pathway presumably triggering DDR and propagating the SASP.

Liu *et al*. (2023b) showed that telomerase (*Terc*) knockout mice similarly had reduced gut microbiota diversity, lipid absorption, intestinal gene expression of a tight junction receptor, and increased inflammation, which suggests gut barrier dysfunction (e.g. Laukoetter *et al*., 2007). However, the microbiota was enriched in Bifidobacteriaceae, which has been reported to reduce gut permeability (Xiao *et al*., 2014), and in Erysipelotrichaceae, which is involved in lipid metabolism (Kaakoush, 2015). While this indicates a beneficial change in the gut microbiome in response to host loss of telomerase or short telomeres, the underlying mechanisms of host regulation of the microbiome are not clear (Liu *et al*., 2023b). Members of Bifidobacteriaceae have also been associated with longer leukocyte TL in humans (Zhang *et al*., 2025b) and with improved gut barrier integrity (Yao *et al*., 2021) in telomerase (*Terc*) knockout mice (Qi *et al*., 2023; Table 1). Members of Erysipelotrichaceae have also been associated with longer DNAmTL in humans with HIV (Singh *et al*., 2024).

The above studies provide experimental support that telomere shortening of the gut tissue has effects on systemic and local intestinal inflammation in the host and on the gut microbiota composition (El Maï *et al*., 2023; Liu *et al*., 2023b), but that these effects may be ameliorated by telomere maintenance. This suggests the possibility of altering replicative senescence by modulating the gut microbiome (Conway & Duggal, 2021; Ghosh *et al*., 2022a) or to treating intestinal disease and dysfunction through gut tissue telomere dynamics. However, in naturally ageing 2‐year‐old wildtype zebrafish, gut‐specific telomerase activation (i.e. telomerase overexpression) had limited effects on ageing (increased cell proliferation and gut lamina propria width, and reduced gut cell senescence) and did not extend lifespan (El Maï *et al*., 2023), suggesting that potential therapeutic applications may be limited to pathogenic conditions where telomere maintenance is impaired (Section II.4). Indeed, associations between telomerase activation and tumorigenesis (Sharma & Chowdhury, 2022) imply that caution is needed in such approaches. Yet, the extent to which these experimental findings in fish can be generalised across tetrapods remains unknown.

### (4) Telomere dynamics under gastrointestinal pathogenic conditions

IBD, which encompasses ulcerative colitis and Crohn's disease, is associated with gut dysbiosis, chronic gastrointestinal inflammation, epithelial damage and immune system dysfunction (Halfvarson *et al*., 2017; Zhang *et al*., 2024). IBD is also associated with increased gut telomere shortening, which has been hypothesised to be due to inflammatory processes leading to higher levels of oxidative stress (Kinouchi *et al*., 1998; O'Sullivan *et al*., 2002; Risques *et al*., 2008; Sienkiewicz *et al*., 2023a). IBD is also associated with dysregulation of telomere‐binding proteins (Da‐Silva *et al*., 2010; Sienkiewicz *et al*., 2023b) and with gut telomerase deficiency (Usselmann *et al*., 2001).

In mice with a mutation in the DNA helicase *Rtel1* gene resulting in short ‘human‐length’ telomeres, the ability of their colonic epithelium to regenerate following treatment with dextran sodium sulphate (DSS), which causes an ulcerative colitis‐like pathology, was reduced (Smoom *et al*., 2023; Zhang *et al*., 2025a), while telomerase‐overexpressing mice were more resistant to a similar ulcerative colitis challenge (Tomás‐Loba *et al*., 2008). In humans, telomere shortening in colonic mucosa is associated with ulcerative colitis [Friis‐Ottessen *et al*., 2014; Risques *et al*., 2011; Tahara *et al*., 2015; Watanabe *et al*., 2022; but see Getliffe *et al*. (2005) who found no association], which may be ameliorated by telomerase activation (Watanabe *et al*., 2022). Conversely, humans with germline mutations in telomere maintenance genes experience higher rates of intestinal inflammation and IBD (Chakravarti *et al*., 2021; Jonassaint *et al*., 2013).

Furthermore, inducing short telomeres in mice and zebrafish activated the oncogenic yes‐associated protein 1 (YAP1) transcription factor in the intestinal epithelium, and this inflammatory pathway similarly caused gut tissue inflammation and IBD (Chakravarti *et al*., 2020; El Maï *et al*., 2023). Gut telomerase reactivation reverted the YAP pathway in zebrafish (El Maï *et al*., 2023) and reduced telomere dysfunction‐induced inflammation in epithelial organoids derived from IBD patients (Chakravarti *et al*., 2020). YAP1 interacts with the YES proto‐oncogene 1 (YES1), which has been negatively genetically correlated with TL alongside other inflammatory biomarkers in a recent human proteomic Mendelian randomisation analysis (Zhao *et al*., 2024). Furthermore, YES1 promotes the formation of epithelial tight junctions (Chen *et al*., 2002). The Hippo pathway, which regulates cell proliferation and apoptosis through activation of YAP1/YES1, could be an important bidirectional molecular link between TL and the environment (Ahi, Panda & Primmer, 2025) including the intestinal microbial environment. For example: YAP1 directly regulates *TERT* transcription in mice and humans; the transcriptional co‐activator of the Hippo pathway, TAZ (transcriptional co‐activator with PDZ‐binding motif), can reduce expression of telomere‐binding genes in cancer cells; and the transcriptional enhanced associate domain (TEAD) transcription factors, which interact with YAP1/TAZ, directly bind to *Drosophila* telomeres (reviewed in Ahi *et al*., 2025). Curiously, a similar (Hippo) signalling pathway has been inferred from *Helicobacter pylori* infection, which activated YAP1 resulting in inflammation‐associated tumorigenesis potentially leading to gastric cancer in mice and human cells (Wu *et al*., 2019). *H. pylori* is associated with telomere shortening and increased telomerase activity leading to gastrointestinal inflammation, increased oxidative damage in gastric mucosa and gastric cancer in humans (Chung *et al*., 2002; Huang *et al*., 2018; Kuniyasu *et al*., 2003; Lee *et al*., 2016). Eradication of this bacterium both restored TL (Aslan *et al*., 2013) and decreased telomerase activity (Chung *et al*., 2002) in humans, suggesting that telomerase activation does not counteract the increased telomere shortening, but may be a critical step in the progression of gastric cancer (Chung *et al*., 2002). Conversely, telomerase deficiency can enhance IBD induced by *Helicobacter mastomyrinus* in mice (Eaton *et al*., 2011).

Antibiotic treatment is often used as an exclusion criterion in epidemiological gut microbiome studies, and little is known about associations between antibiotic use and TL. Glapa‐Nowak *et al*. (2021) reported shorter telomeres in humans treated with antibiotics in the sampling period, but without investigating the gut microbiota. Chen *et al*. (2022) found no effect of maternal antibiotic use on child TL. Chakravarti *et al*. (2020) found that antibiotic treatment of telomerase (*Tert*) knockout mice reduced intestinal inflammation induced by telomere dysfunction, but telomerase‐active controls were not subjected to the same treatment. Future studies could utilise antibiotic treatment to explore functional links between changes in the gut microbiome and telomere dynamics.

Telomere shortening can potentially anticipate the (early) onset of age‐related diseases across the organism, including cancer, and inflammatory and neurodegenerative conditions. Telomere dysfunction and both too short and too long telomeres can initiate gastrointestinal neoplasia and alter the tumour microenvironment (LaBella *et al*., 2024; Zhang *et al*., 2025c). Furthermore, several microbes are known to influence the development of cancer (Thomas *et al*., 2017) including repression of telomerase (Chung *et al*., 2002; Liu *et al*., 2019a). Thus, investigating whether the microbiota–telomere crosstalk interacts with the oncobiota (i.e. the community of cancer cells; Thomas *et al*., 2017) may reveal novel insights into gastrointestinal carcinogenesis (e.g. Huang *et al*., 2018).

Harnessing the bidirectional gut–microbiome–telomere interactions for therapeutic interventions could mitigate age‐related disorders and pathologies or pave the way for emerging treatments of telomere‐mediated gastrointestinal disorders. For example, acute graft‐versus‐host disease is a frequent complication of unrelated donor bone marrow transplantation (allogenic hematopoietic stem cell transplantation) that often manifests in the intestines, where donor immune cells attack host tissue (Hummel *et al*., 2015). This gut tissue damage results in increased cell proliferation, dramatic enterocyte telomere shortening and higher mortality (Hummel *et al*., 2015). However, the observation that long pre‐transplantation recipient TL appears to buffer against such intestinal injury (Myllymäki *et al*., 2020) suggests that intestinal TL could be a clinically relevant target for such conditions, but how TL influences intestinal regeneration is not well understood (Montgomery *et al*., 2011). The microbial causes of dysbiosis are often not well known but are associated with weakened host control over the gut microbiota (Lee, Tsolis & Bäumler, 2022). Therefore, the host gut physiology may represent a more tractable approach to remediating gut dysbiosis, rather than manipulation of the microbiota, in future translational research (Lee *et al*., 2022).

## III. CHALLENGES TO INVESTIGATING MICROBIOTA–TELOMERE INTERACTIONS

Telomeres are involved in several molecular and cellular pathways through which changes in TL may affect the gut microbiota, but such potential regulatory mechanisms have rarely been investigated in their entirety. For example, telomere shortening can regulate the expression of sirtuins (Amano *et al*., 2019) – signalling proteins that interact with the gut microbiota and regulate metabolism (Zhang *et al*., 2018). Non‐coding RNAs, such as microRNAs with gene regulatory functions, are involved in TL regulation (Rossi & Gorospe, 2020), and telomere shortening may affect the expression of several microRNAs presumably involved in tumour progression (Castro‐Vega *et al*., 2013). MicroRNAs are emerging as important mediators of host–microbe interactions where they also play a role in regulating gut barrier functions, providing another bidirectional pathway of microbiota‐induced effects on host physiology (Virtue *et al*., 2019) as well as host control of the gut microbiota (Liu *et al*., 2016; Pepke, Hansen & Limborg, 2024b). However, such mechanisms have not been investigated within controlled experiments of telomere dynamics manipulation.

There are several factors that may indirectly link gut microbiota with telomere dynamics and thus may confound or complicate investigating their direct causal relationship. Dietary components can influence both the gut microbiome (David *et al*., 2014) and telomere dynamics (O'Callaghan *et al*., 2012; Zhu *et al*., 2025) through independent or similar pathways that are difficult to separate (Niu *et al*., 2021). However, diets that support a diverse and healthy gut microbiome may also contribute to maintaining TL (Anusha & Negi, 2024; Galiè *et al*., 2020) through reduced oxidative stress (Qiao *et al*., 2013). Certain gut bacteria are involved in dysregulation of metabolism, such as insulin resistance (Takeuchi *et al*., 2023), which is linked to accelerated telomere shortening (Demissie *et al*., 2006) or short telomeres (Verhulst *et al*., 2016) and adipokine imbalance (Broer *et al*., 2014). TL is also regulated by metabolic processes that can be independent of oxidative stress (Casagrande & Hau, 2019). Such processes may involve the gut microbiota, e.g. through the production of microbial metabolites such as thymidine (Xiong *et al*., 2021) that are key components of telomere maintenance (Mannherz & Agarwal, 2023).

Other poorly explored indirect links between the microbiome and telomere dynamics include causes and consequences of organismal growth. For instance, the gut microbiota has been shown to regulate insulin‐like growth factor 1 (IGF‐1) and growth hormone production thereby affecting the somatotropic axis and growth (Robertson *et al*., 2019). Telomeres shorten during growth in many species, potentially limiting cell proliferation capacity (Pepke *et al*., 2022c), but no studies have yet shown if changes in TL and (microbial) metabolites are linked during growth. Furthermore, TL is a highly polygenic trait (Burren *et al*., 2024; Codd *et al*., 2021; Pepke *et al*., 2022a; Taub *et al*., 2022) and genes involved in the regulation of TL or telomere maintenance may be pleiotropic, i.e. affecting multiple other traits, such as inflammatory responses (Liu *et al*., 2023a) or oxidative‐stress‐inducing processes (Pepke *et al*., 2022a). Such pleiotropic effects could underlie genetic correlations between telomere dynamics and microbial traits, as indicated by preliminary results from Lei *et al*. (2022a) showing correlations between TL and intestinal *Sutterella* species in humans, and from Onarman Umu *et al*. (2024) showing correlations between telomere maintenance genes and *Subdoligranulum* species in pigs. The interconnectedness of these interactions – and our observation that the microbiome and telomere dynamics apparently can be influenced by the same factors – suggest that integrated (holo‐omic) analyses and experimental approaches are required to unravel these links further.

Animal models and experiments have played an important role in studies on interactions between telomeres and microbiota especially under pathogenic conditions. However, while for example killifish (Section II.2), zebrafish (Section II.3) and other teleosts have short telomeres similar to humans (Henriques & Ferreira, 2024; Poeschla & Valenzano, 2020), highly inbred, domesticated strains of laboratory mice have extremely long telomeres compared to their wild counterparts (Hemann & Greider, 2000) that might lengthen with age (Ilmonen, Kotrschal & Penn, 2008). This may be due to effects of artificial selection or captivity on TL (Pepke & Eisenberg, 2022), questioning the generalisability of telomere dynamics in traditional laboratory mice. Laboratory domestication and captivity conditions may also have left an impact on the gut microbiome composition of mice (Bowerman *et al*., 2021) and other species (Alberdi, Martin Bideguren & Aizpurua, 2021). However, mice can readily be engineered to lack telomerase and thus have short human‐like telomeres and show early onset of age‐associated degenerative diseases and telomere dysfunction (Kang *et al*., 2018). Such models may reveal further insights into the bidirectionality of interactions between telomeres and microbiota.

## IV. REVISITING THE MICROBIOTA–GUT–BRAIN AXIS

The microbiota–gut–brain axis refers to the bidirectional communication between the microbiota, intestinal tissue and brain that involves several pathways including the enteric nervous system, microbial metabolites and the immune system (Cryan *et al*., 2019). Disturbance of the microbiota–gut–brain axis is associated with dysregulation of the hypothalamic–pituitary–adrenal (HPA) axis (Farzi, Fröhlich & Holzer, 2018). The HPA axis is involved in stress response through e.g. stress hormone (cortisol) release, and it can be activated by telomere shortening (measured in blood cells; Tomiyama *et al*., 2012). Furthermore, TL apparently responds to psychological stress in humans (Epel *et al*., 2004), and correlative evidence suggests that this occurs through effects of higher oxidative stress and lower telomerase expression (Epel *et al*., 2004; Lin & Epel, 2022). Gut microbiota‐induced oxidative stress involved in neurodegeneration (Shandilya *et al*., 2022) may act in coordination or in parallel with changes in TL (Pousa *et al*., 2021; Xu *et al*., 2023) in the gut (a highly proliferative tissue) or the brain (low proliferation). It has therefore been suggested that telomere dynamics should be a focus of future research on mechanisms underlying disorders of the microbiota–gut–brain axis (Bazaz *et al*., 2021; Manchia *et al*., 2020). Irritable bowel syndrome (IBS) is a gastrointestinal dysfunction associated with psychiatric conditions (anxiety and depression) that has recently been recognised as a microbiota–gut–brain axis disorder involving changes in brain functions (Mayer, Ryu & Bhatt, 2023). Although the primary symptom triggers of IBS are not well known, patients with IBS have shorter leukocyte telomeres (Wang *et al*., 2023; Zhang *et al*., 2022a). Such associations are important if telomere dynamics can manifest as prolonged consequences of gut dysbiosis or psychiatric conditions. A study using FMTs in Brandt's voles (discussed in Section II.2) linked crowding stress to shorter brain TL and to gut microbiome differences, which could also induce telomere shortening (Xu *et al*., 2023). However, we found no empirical studies including microbiome data that linked telomere dynamics with microbiota–gut–brain disorders in humans.

## V. ARE TELOMERE DYNAMICS MODULATORS OF ADAPTIVE HOST–MICROBE EVOLUTION?

Understanding the crosstalk between the gut microbiome and telomere dynamics extends beyond investigating mechanisms of ageing, development and disease. Both telomeres and the microbiome may independently constitute – or be associated with – components of host fitness in humans (Wang *et al*., 2018; Wilmanski *et al*., 2021) and in wild animals (Suzuki, 2017; Wilbourn *et al*., 2018). We hypothesise that telomere‐mediated microbiome–host interactions could influence rapid evolutionary adaptations of the holobiont physiology. However, the magnitude and mode of transgenerational inheritance of both telomere dynamics (Pepke *et al*., 2022a) and of the gut microbiome (Morris & Bohannan, 2024; Ryu & Davenport, 2022) is debated and may vary considerably across host species and bacterial taxa. Furthermore, both can be influenced by similar environmental exposures (Bestion *et al*., 2017; Chatelain *et al*., 2020; Pepke *et al*., 2022b), and it is possible that the stress responses of the gut microbiome (Houtz, Taff & Vitousek, 2022; Noguera *et al*., 2018) and of gut telomere dynamics (Lin & Epel, 2022; Pepper, Bateson & Nettle, 2018) are interacting. For example, higher temperatures resulted in longer TL in the gut, but not in other tissues including muscle, heart and liver in African clawed frogs (*Xenopus laevis*; Burraco, Metcalfe & Monaghan, 2025).

One challenge with inferring an adaptive role of telomere dynamics in mediating microbiota–host adaptations, is that we still know little about the (physiological or life‐history) costs of telomere maintenance (Monaghan, 2024; Pepke *et al*., 2023a). Such costs are probably not limited to the energy required to maintain telomeres (which may indeed be negligible; Young, 2018), but could include costs associated with increased cancer risk, the adaptive regulatory role of TL, or non‐canonical roles of telomerase activity (DeBoy *et al*., 2023; Pepke & Eisenberg, 2022; Smith *et al*., 2021; Young, 2018). Indeed, TL may reflect cumulative, experienced somatic impact of an individual's exposome, i.e. TL may be a marker of individual condition and physiological state (Pepper *et al*., 2018) without implying strong causal roles of TL on fitness or the ageing processes in wild animal populations (Boonekamp *et al*., 2013; Pepke *et al*., 2022b). However, the causal role of telomere dynamics is an ongoing debate, particularly in the wild, where causality can be difficult to disentangle (Tobler *et al*., 2022; Young, 2018). In a conceptually similar manner, the individual microbiome composition reflects life experiences and environmental influences (Si *et al*., 2022; Wilmanski *et al*., 2021) although there may be a considerable genetic component that varies across species (Litichevskiy *et al*., 2025; Ryu & Davenport, 2022). Long‐term studies across multiple generations are necessary to investigate, for example, the variance in telomere dynamics that can be explained by the gut microbiome (i.e. microbiability; Morris & Bohannan, 2024), in addition to potential host genome–microbiome interactions shaping TL (i.e. holobiability; Venegas *et al*., 2023). Such studies are needed to test if telomere dynamics are modulators of adaptive host–microbe evolution.

## VI. LEVERAGING WHOLE GENOME SEQUENCING FOR TELOMERE RESEARCH

**Fig. 3:**
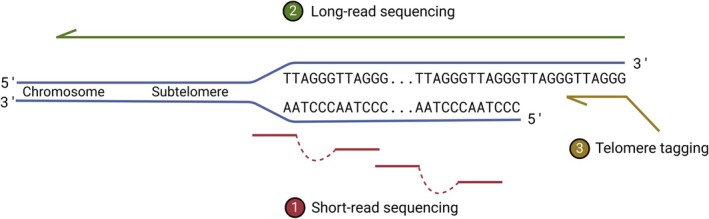
Illustration of telomere sequencing methods based on (1) short‐read sequencing, shown here as read‐pairs; (2) long‐read sequencing spanning the telomere, subtelomere and chromosome; (3) various methods that tag and amplify specific functional telomeres prior to sequencing (see Table 2).

Long repetitive regions of the genome, such as telomeres, are structurally challenging to resolve and telomeric sequences are often discarded from whole genome sequencing (WGS) data (Miga & Sullivan, 2021; but see Choo *et al*., 2023). However, it is possible to estimate average TL *in silico* with reasonable accuracy from short‐read WGS data by simply counting the canonical telomere repeats (5′‐TTAGGG‐3′, Fig. 3) and normalising by genome coverage (Castle *et al*., 2010; Ding *et al*., 2014; Parker *et al*., 2012). A threshold number of intact TTAGGG repeats within reads can be applied to reduce the likelihood of including interstitial telomere sequences (Ding *et al*., 2014). It is also possible to estimate average TL from whole exome sequencing data, which primarily targets protein‐coding sequences, but the accuracy is reduced compared to WGS (see Feuerbach *et al*., 2019; Hu, Ghandi & Huang, 2021). Various bioinformatic tools have recently been designed to determine telomeric content or absolute TL with different approaches to alignment and to discriminate between interstitial telomeric repeats and functional telomeres, and to account for sequencing errors and differences in read lengths (Table 2). Such methods have often been overlooked, perhaps due to cost and technical difficulties, when considering TL measurement methodologies or designing research studies, but they are largely congruent with laboratory‐based methods that estimate average TL (Lee *et al*., 2017).

**Table 2:** Chronological list of 26 bioinformatic tools, pipelines and sequencing approaches (including pre‐sequencing enrichment methods) that can be used to estimate telomere length (TL) from short‐ or long‐read whole genome sequencing (WGS) data including a brief method description (see also Fig. 3) and recent examples of their use with empirical data.

Tool	Reference	Method overview	Developed for long‐read data?	Examples
motif_counter	Conomos *et al*. (2012)	Counts a motif sequence repeated a specified number of times from aligned reads.	No	Lee *et al*. (2017)
TelSeq	Ding *et al*. (2014)	Counts the number of telomeric reads (containing at least seven TTAGGG repeats) from aligned reads and normalises for sequencing depth by GC‐composition.	No	Huang *et al*. (2018); Lee *et al*. (2017); Sung *et al*. (2023); Taub *et al*. (2022); Wang *et al*. (2022); Burkert *et al*. (2024); Burren *et al*. (2024); Hu *et al*. (2021)
k‐Seek	Wei *et al*. (2014); Wei *et al*. (2018)	Identifies and quantifies simple tandem repeats (k‐mers) from short‐read data. Normalising by GC content improves TL estimates.	No	Choi *et al*. (2021); Zavala‐Paez, Holliday & Hamilton (2023)
Computel	Nersisyan & Arakelyan (2015)	Aligns short‐read sequences to a telomeric reference sequence (‘index’) and counts reads normalised by coverage.	No	Lee *et al*. (2017); Sung *et al*. (2023); Taub *et al*. (2022); Zavala‐Paez *et al*. (2023)
TeloPCR‐seq	Bennett *et al*. (2016)	Targeted amplification and sequencing of telomeres in fission yeast *Schizosaccharomyces pombe*.	Yes	–
Telomerecat	Farmery *et al*. (2018)	Counts telomere reads normalised by reads at the subtelomeric boundary regions allowing for aneuploidy (e.g. cancers).	No	Machado *et al*. (2022)
TelomereHunter	Feuerbach *et al*. (2019)	Alignment‐based determination of telomeric content and variants designed for matched tumour and control samples. Also validated using whole genome bisulfite and whole exome sequencing data.	No	Sung *et al*. (2023)
TLD (telomere length determination)	Reed *et al*. (2021)	Uses single‐molecule real‐time (SMRT) sequencing data to estimate TL based on a 200 bp window within a telomere motif content threshold.	Yes	–
edgeCase	Grigorev *et al*. (2021)	Aligns long‐reads to a telomere‐annotated human reference genome for chromosome‐specific *de novo* telomeric motif discovery.	Yes	Luxton *et al*. (2020)
Telogator/Telogator2	Stephens *et al*. (2022); Stephens & Kocher (2024)	Similar methodology to edgeCase, enabling estimating chromosome‐arm‐specific TL.	Yes	–
qmotif	Holmes *et al*. (2022)	Efficient method to identify candidate ‘telomeric’ reads and subsequently count telomeric repeats.	No	Lee *et al*. (2017); Wang *et al*. (2022)
TRIP (telomeric repeats identification pipeline)	Yihang *et al*. (2022)	Identifies telomeric repeat motifs *de novo* from short‐read WGS data.	No	Zavala‐Paez *et al*. (2023)
TeloTag	Sholes *et al*. (2022)	Method to tag telomere ends prior to Nanopore sequencing. Reads are extracted using TideHunter (Gao *et al*., 2019).	Yes	Karimian *et al*. (2024)
seqtk telo	Li (2023)	Rapid identification of all telomere repeats in sequences in FASTA/Q format.	No	Fang & Edwards (2024)
ChArmTelo (chromosome arm‐level telomeres)	Guo *et al*. (2023)	Estimates TL from 10X linked‐read WGS data by assigning molecules with the same barcode to chromosome arms after extracting reads using TelomereHunter.	No	–
NanoTelSeq/Telomere Analyzer	Smoom *et al*. (2023)	Telomere end‐specific ligation is used to enrich long‐read Nanopore sequencing of telomeres.	Yes	Smoom *et al*. (2025)
Telomap	Tham *et al*. (2023)	Method to enrich telomeres prior to PacBio HiFi sequencing.	Yes	–
Y^ea^ISTY	D'Angiolo *et al*. (2023)	Estimates average TL, interstitial telomeric content and Y′ copy number in yeast.	No	–
Telofinder	O'Donnell *et al*. (2023)	Determines location and size of telomere sequences in yeast genome assemblies.	Yes	–
Telo‐seq	Schmidt *et al*. (2024)	Method to enrich telomeres prior to Nanopore sequencing.	Yes	–
Telometer	Sanchez *et al*. (2024)	Estimates TL after alignment of WGS or telomere‐enriched long‐read data to the human reference genome.	Yes	–
TelSize	Tan *et al*. (2024)	Estimates chromosome‐specific TLs from noisy long‐read data using a moving window approach.	Yes	–
Telomere Profiling	Karimian *et al*. (2024)	Method to enrich telomeres using TeloTag prior to sequencing using ONT MinION.	Yes	–
TeloNum	Colt *et al*. (2024)	Pipeline to estimate TL directly from raw (unprocessed) ONT and PacBio long reads.	Yes	Lynch *et al*. (2025)
TECAT (telomere end chromosome assaying tool)	Reed *et al*. (2025)	Telomere motif discovery, chromosome‐specific TL determination by reference sequence alignment, and interactive visualisation.	Yes	–
Topsicle	Nguyen & Choi (2025)	Estimates TL from long‐read WGS data using k‐mer and change point detection analysis to determine the telomere–subtelomere boundary.	Yes	–
TARPON (telomere analysis and research pipeline optimized for Nanopore)	Deimler *et al*. (2026)	Pipeline optimised for Nanopore sequencing analysis of chromosome‐specific TLs. Includes a user‐friendly graphical interface through EPI2ME.	Yes	–

The advent of long‐read sequencing, such as Pacific Biosciences (PacBio) High‐Fidelity (HiFi) and Oxford Nanopore Technologies (ONT) sequencing (Wang *et al*., 2021b) allows unprecedented repeat resolution by generating and mapping continuous sequences spanning long regions (Tan *et al*., 2022; van Dijk *et al*., 2023). This allows sequencing through telomeres to generate telomere‐to‐telomere reference genomes (Nurk *et al*., 2022) and resolving TL for individual chromosome arms with increased accuracy (Sholes *et al*., 2022; Stephens & Kocher, 2024). Another advantage of long‐read sequencing is library preparation without amplification, which retains the methylation signal allowing analyses of the epigenetic regulation of telomere dynamics (e.g. Maeda *et al*., 2021; Shim *et al*., 2024). Although the costs of (in particular long‐read) DNA sequencing may still hinder its use as a dedicated TL test compared to other methods (Ferrer, Stephens & Kocher, 2023), modified protocols to tag, enrich or purify full‐length telomere DNA prior to sequencing (Fig. 3; Table 2) hold promise as high‐throughput approaches (without requiring sequencing of whole genomes) in clinical settings (Schmidt *et al*., 2024; Tham *et al*., 2023). However, such methods often require large amounts of genomic DNA, they remain labour‐intensive both pre‐ and post‐sequencing (Karimian *et al*., 2024) and are consequently still in the process of being further developed and evaluated against appropriate controls. In Table 2 we provide an overview of bioinformatic tools, pipelines and sequencing approaches that have been developed to estimate TL from short‐ or long‐read WGS data.

Importantly, sequencing data allow simultaneous genomic and TL analyses. For instance, Taub *et al*. (2022) used the bioinformatic software TelSeq (Table 2; Ding *et al*., 2014) to estimate TL from WGS data from a large cohort study (TopMed) across >109,000 humans and identified loci associated with TL which largely matched those identified by qPCR‐based TL measures (Codd *et al*., 2021). Burren *et al*. (2024) used a joint metric of qPCR and TelSeq TL estimates which increased the contribution of genetic variance in explaining TL across >460,000 humans (UK Biobank) and identified additional rare genetic variants associated with TL, suggesting that each method captures complementary information.

Due to the costs of WGS it is less likely to be used in experiments where the main aim is TL estimation, but existing data from epidemiological and large‐cohort studies, population‐scale biobanks (Burren *et al*., 2024; Taub *et al*., 2022), large interspecific WGS projects (Rhie *et al*., 2021), pangenomic (Fang & Edwards, 2024) or hologenomic studies combining host genomics and metagenomics (Leonard *et al*., 2024; Zhang *et al*., 2024) represent largely untapped resources and unprecedented opportunities for telomere research both within and across species. The study of host‐associated microbiomes has similarly been transformed with the development of high‐throughput and cost‐effective sequencing technologies, such as amplicon sequencing and more recently, non‐targeted shotgun and long‐read metagenomics (Kim, Pongpanich & Porntaveetus, 2024). In principle, it is possible to estimate (gut‐derived) TL from the host DNA that is normally filtered from metagenomic sequencing data, but to our knowledge this approach has not yet been tested. Integrating sequencing data into telomere studies and *vice versa* will provide deeper insights into the host–microbiome interactions highlighted in this review.

## VII. FUTURE PERSPECTIVES: THE BEGINNING OF UNDERSTANDING THE END

New mechanisms and pathways of regulating TL are still being uncovered (Takai *et al*., 2024). These include a protein that stimulates telomere trimming (Li *et al*., 2017), which may be a tumour suppressor mechanism (Schmutz *et al*., 2020); other novel telomere binding proteins (Braun *et al*., 2023); the complex role of telomere regulatory shelterin proteins (Vinayagamurthy *et al*., 2023; Wolf & Shalev, 2023); telomere elongation by extracellular telomere vesicle transfer (Lanna *et al*., 2022); telomerase‐mediated ROS protection (Barcenilla *et al*., 2023); telomere‐dysfunction‐induced oxidative stress (von Zglinicki, 2024); and the epigenetic regulation of telomere dynamics (Burkert *et al*., 2024; Shim *et al*., 2024). Although these interactions are not well established, some of these mechanisms are revealing causal effects of telomere dynamics on host physiology. Furthermore, TL is involved in (reversible) silencing of genes near telomeres (telomere positioning effect; Baur *et al*., 2001), but telomeres can also regulate genes over longer distances by looping back on the chromosome (Kim & Shay, 2018). Thus, telomere shortening can have functional consequences without reaching critically short TLs.

Telomeres are transcribed into long telomeric repeat‐containing RNA (TERRA), which were presumed to be non‐coding, but may be important in maintaining telomeres and thus regulating TL through a feedback loop (Montero *et al*., 2016). However, the conventional understanding that telomeres are not translated was recently challenged by Al‐Turki & Griffith (2023) showing that they encode two simple dipeptide proteins that are thought to be increasingly translated from TERRA when telomeres become critically short. The authors speculated that these proteins could trigger inflammation. The biological significance of this discovery is not yet clear, but it provides a new route of TL‐dependent signalling. The possibility of extracellular release of (anti‐inflammatory) telomeric DNA sequences (Bonafè *et al*., 2020; Storci *et al*., 2019) may be another exciting but little explored mechanism of host modification of the intestinal environment. Furthermore, long‐read sequencing has (*de novo*) identified individual and chromosome‐specific nucleotide variation in the largely conserved 5′‐TTAGGG‐3′ telomere repeats with yet unknown consequences or functions in humans (Grigorev *et al*., 2021; Tham *et al*., 2023). These and other studies point to several non‐canonical roles of telomeres (Vinayagamurthy *et al*., 2023) and of telomerase (Smith *et al*., 2021) that are likely to change our understanding of the causes and consequences of telomere dynamics.

**Fig. 4:**
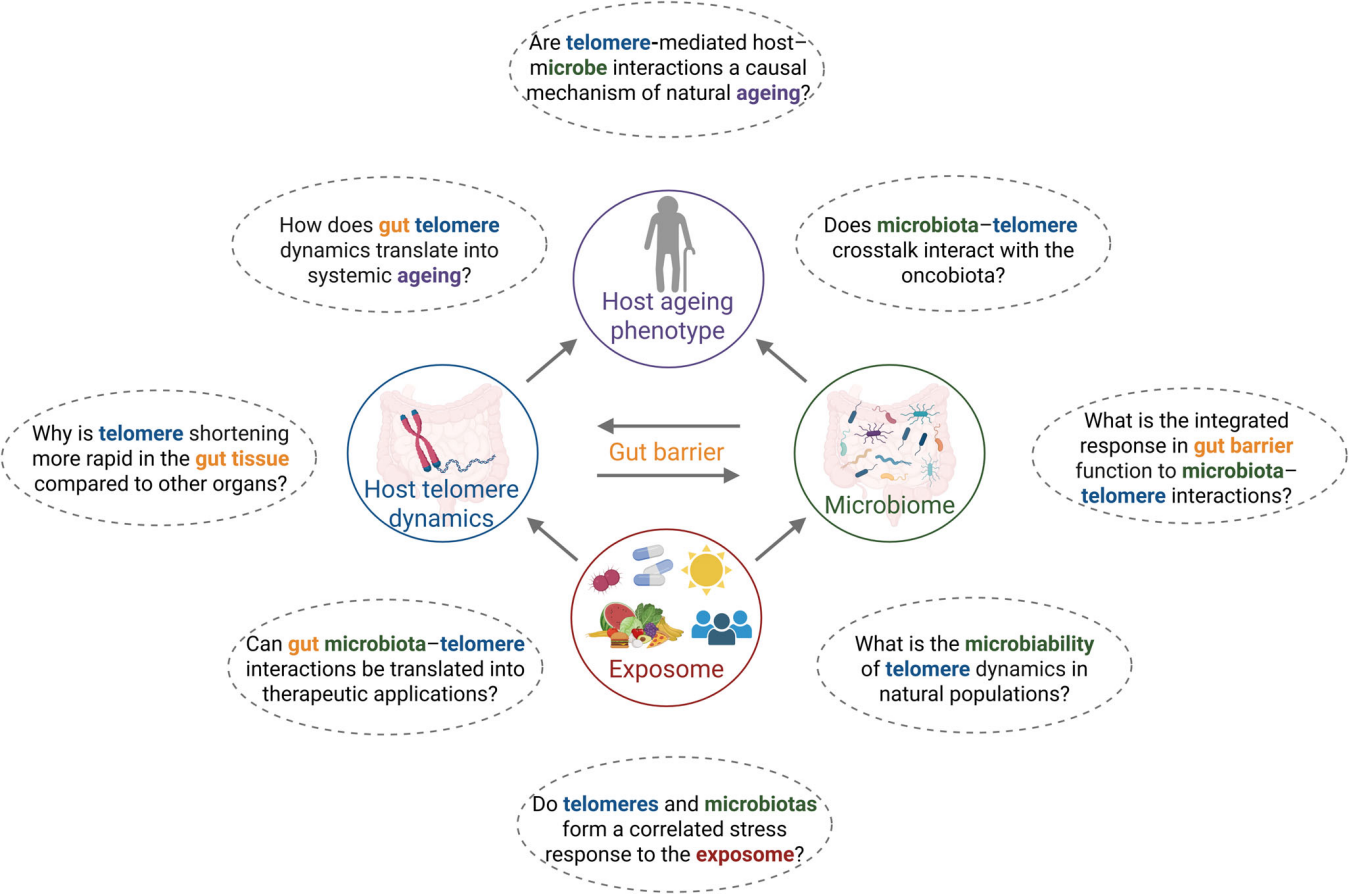
Interactions between components of the gut microbiota (green) and host telomere dynamics (blue), which are critical in maintaining gut barrier integrity (orange), are both influenced by cumulative environmental exposures (exposome, red) and shape the individual ageing phenotype (purple). Arrows indicate directions of causality. Questions identified in this review with the intention to guide the field forward are summarised in dashed ovals.

The influences of the gut microbiome on the ageing process could be mediated extensively by telomere dynamics (Fig. 1). The large heterogeneity in study designs, results and types of interactions reviewed here largely prevents assessments of publication bias or small study effects (Munn *et al*., 2018), but few studies reported null results (see Boehme *et al*., 2021; Getliffe *et al*., 2005; Gianesin *et al*., 2016; Lin *et al*., 2024a; Makarenko *et al*., 2019; Plancade *et al*., 2019; Ravindran *et al*., 2024; Shijimaya *et al*., 2024; Table 1). As many of the cellular or molecular pathways underlying telomere–microbiome interactions are not well known (Fig. 4), explorative studies are still needed. However, future studies should explicitly account for differences between healthy and unhealthy ageing (Ghosh *et al*., 2022a,b) when investigating links between the microbiome, telomere dynamics, and ageing phenotypes. This distinction may be important for disentangling causal mechanisms from correlative patterns and for identifying specific microbial signatures predictive of healthy ageing.

How telomere dynamics interact with microbially mediated stress responses remains little explored, but here we suggest a framework for understanding telomere dynamics at the holobiont level, linking microbiomes to host traits and phenotypes. The early onset and severity of telomere shortening on the intestinal tissue may be partly attributed to its high cell proliferation rate. While this disproportionality compared to other rate‐limiting organs is not yet well understood (Fig. 4), the intestinal tract stands out as a promising focal tissue for unravelling intimate host–microbiota interactions and within‐body mosaics of ageing through a hologenomic framework (Bordenstein & Theis, 2015; Kawamoto & Hara, 2024; Pepke *et al*., 2024a; Salazar *et al*., 2023; Zhang *et al*., 2024). Other tissues with a prevailing microbiota, such as the skin (Duarte *et al*., 2024) and lungs (Kang *et al*., 2018; Liu *et al*., 2018), may interact similarly with telomere dynamics within those tissues, but this remains poorly explored.

## VIII. CONCLUSIONS

(1) Gut microbiome composition and activity is often associated with host telomere dynamics through ROS‐generating processes, microbial metabolites and interactions with gut barrier integrity. Longer telomeres tend to be associated with higher microbiota diversity and with anti‐inflammatory and probiotic species. Shorter telomeres tend to be associated with lower microbiota diversity and with proinflammatory and pathogenic species.
(2) Telomerase deficiency induces increases in pathogenic species and a dysbiotic gut microbiome, although there is some evidence of adaptive increases in potentially beneficial species. Manipulating gut tissue telomere dynamics can affect gut microbiomes through changes in gut physiology, barrier integrity, gene expression, and release of SASP factors, suggesting that interactions between the gut microbiome and host telomere dynamics are bidirectional.
(3) Microbiota–telomere dynamics link inflammation and gut dysbiosis, and these interactions are emerging as important in maintaining intestinal homeostasis, which influences host health and disease.
(4) Evidence for telomere‐mediated microbiome–host interactions is based on FMT experiments, intestinal organoid models, and telomerase manipulation experiments mainly in vertebrate laboratory animals. Observational studies on humans and a few non‐model animals also provide evidence for associations between telomere and microbiome dynamics. Demonstrations across different species suggest that such interactions are evolutionarily conserved in many vertebrates.
(5) WGS data represents an unexploited resource for telomere research. Furthermore, recent advances in WGS capacities and bioinformatic tools will be instrumental in future systems biology approaches that integrate telomeres as part of high‐throughput data‐analysis frameworks.
(6) The associations and experimental manipulations of the crosstalk between microbiotas and telomere dynamics outlined here are mainly investigated using cross‐sectional or single time‐point interventions. Few longitudinal studies have investigated how these hallmarks of ageing interact during the lifespan, and such studies may be challenged by multiple confounders. However, an understanding of whether telomere dynamics are causal mediators of gut microbiota–host interactions is crucial to adopting a new holobiont theory of ageing.

## AUTHOR CONTRIBUTIONS

M. L. P. conceived the study, compiled literature and drafted the manuscript with contributions from S. B. H. and M. T. L. All authors revised and approved the manuscript.

## Supporting information


**Fig. S1.** PRISMA chart of the literature search process.

## Data Availability

Data sharing not applicable to this article as no datasets were generated or analysed during the current study.
