# Supplementary material for: The ageing holobiont: crosstalk between telomere dynamics, oxidative stress and the gut microbiome
Source: Biol Rev Camb Philos Soc. 2026 Feb 27;101(4):1877–903. doi: 10.1002/brv.70152 (PMC13326800; doi:10.1002/brv.70152)
Supplement: Supplementary file 1 — Fig. S1. PRISMA chart of the literature search process. [file BRV-101-1877-s001.docx]

**Supporting Information**

**Identification of studies *via* databases**

Duplicates removed (*N* = 45)

Articles identified from *Web of Science* and *Scopus* combined (*N* = 195)

Additional articles identified from forward and backward reference searches etc. (*N* = 43)

**Identification**

Articles excluded following criteria (*i*), (*ii*) or (*iii*) (*N* = 114)

Articles screened (*N* = 193)

**Screening**

Associations between telomere dynamics and the gut microbiome (*N =* 15)

Articles included in review

(Table 1, *N* = 79)

Telomere dynamics and faecal microbiota transplantation (*N =* 5)

**Included**

Gut microbiomes of telomerase knockout models (*N =* 18)

Telomere dynamics under gastro-intestinal pathogenic conditions (*N* = 23)

Experimental treatments affecting both telomere and microbiota dynamics (*N =* 20)

**Fig. S1.** PRISMA chart of the literature search process. Studies from the systematic search were individually screened to assess whether they (*i*) include analyses of both the gut microbiome and host telomere dynamics in any tissue, (*ii*) are based on empirical data collected *in vivo* or *in vitro*, and (*iii*) reported how and where telomere length was measured (see Section I.3). Two studies occur in two topic categories.
